# HPV.edu study protocol: a cluster randomised controlled evaluation of education, decisional support and logistical strategies in school-based human papillomavirus (HPV) vaccination of adolescents

**DOI:** 10.1186/s12889-015-2168-5

**Published:** 2015-09-15

**Authors:** S. Rachel Skinner, Cristyn Davies, Spring Cooper, Tanya Stoney, Helen Marshall, Jane Jones, Joanne Collins, Heidi Hutton, Adriana Parrella, Gregory Zimet, David G. Regan, Patti Whyte, Julia M. L. Brotherton, Peter Richmond, Kirsten McCaffrey, Suzanne M. Garland, Julie Leask, Melissa Kang, Annette Braunack-Mayer, John Kaldor, Kevin McGeechan

**Affiliations:** Paediatrics and Child Health, Faculty of Medicine, University of Sydney, Locked Bag 4001, Westmead, Sydney, NSW 2054 Australia; CUNY School of Public Health, City University New York, New York City, NY USA; Vaccine Trials Group, Telethon Kids Institute, Perth, WA Australia; Vaccinology & Immunology Research Trials Unit, Women’s & Children’s Hospital, Adelaide, South Australia Australia; Pediatrics & Clinical Psychology, Indiana University, Indianapolis, IN USA; The Kirby Institute, Faculty of Medicine, UNSW Australia, Sydney, NSW Australia; Deakin University, SRC Population Health, Deakin Health Economics, Melbourne, VIC Australia; National HPV Vaccination Program Register, VCS, and School of Population and Global Health, University of Melbourne, Melbourne, Victoria Australia; School of Public Health, University of Sydney, Sydney, NSW Australia; Women’s Centre for Infectious Diseases, The Royal Women’s Hospital, Melbourne, VIC Australia; Westmead Clinical School, University of Sydney, Sydney, NSW Australia; School of Population Health, University of Adelaide, Adelaide, SA Australia; School of Paediatrics and Child Health, University of Western Australia, Perth, WA Australia

## Abstract

**Background:**

The National Human Papillomavirus (HPV) Vaccination Program in Australia commenced in 2007 for females and in 2013 for males, using the quadrivalent HPV vaccine (HPV 6,11,16,18). Thus far, we have demonstrated very substantial reductions in genital warts and in the prevalence of HPV among young Australian women, providing early evidence for the success of this public health initiative. Australia has a long history of school-based vaccination programs for adolescents, with comparatively high coverage. However, it is not clear what factors promote success in a school vaccination program. The HPV.edu study aims to examine: 1) student knowledge about HPV vaccination; 2) psycho-social outcomes and 3) vaccination uptake.

**Methods/Design:**

HPV.edu is a cluster randomised trial of a complex intervention in schools aiming to recruit 40 schools with year-8 enrolments above 100 students (approximately 4400 students). The schools will be stratified by Government, Catholic, and Independent sectors and geographical location, with up to 20 schools recruited in each of two states, Western Australia (WA) and South Australia (SA), and randomly allocated to intervention or control (usual practice). Intervention schools will receive the complex intervention which includes an *adolescent intervention* (education and distraction); *a decisional support tool* for parents and adolescents and *logistical strategies* (consent form returns strategies, in-school mop-up vaccination and vaccination-day guidelines). Careful process evaluation including an embedded qualitative evaluation will be undertaken to explore in depth possible mechanisms for any observed effect of the intervention on primary and secondary outcomes.

**Discussion:**

This study is the first to evaluate the relative effectiveness of various strategies to promote best practice in school-based vaccination against HPV. The study aims to improve vaccination-related psychosocial outcomes, including adolescent knowledge and attitudes, decision-making involvement, self-efficacy, and to reduce fear and anxiety. The study also aims to improve school vaccination program logistics including reduction in time spent vaccinating adolescents and increased number of consent forms returned (regardless of decision). Less anxiety in adolescents will likely promote more efficient vaccination, which will be more acceptable to teachers, nurses and parents. Through these interventions, it is hoped that vaccination uptake will be increased.

**Trial registration:**

Australian and New Zealand Clinical Trials Registry, ACTRN12614000404628, 14.04.2014.

**Electronic supplementary material:**

The online version of this article (doi:10.1186/s12889-015-2168-5) contains supplementary material, which is available to authorized users.

## Background

With several countries having now implemented HPV vaccination programs, data on uptake are beginning to emerge. Although the age range for comparison varies internationally, there is a vast difference in HPV vaccine uptake across these countries, from very high—86.7 % for 3 doses in adolescent females of the target age in the UK (2013–2014) [1]—to very low—37 % for 3 doses among the adolescent age group in the US [2]. Uptake needs to be consistently high for a comprehensive reduction in HPV disease burden to be achieved at a population-level. In particular, if 3-dose vaccination coverage can be increased from 67 to 90 %, models predict that the long-term reduction in incident infection will be increased from 76 to 95 % [3]. In general, school-based programs tend to have higher coverage than non-school based programs [4].

The National HPV Vaccination Program in Australia commenced school delivery in April 2007 for girls and February 2013 for boys, using the quadrivalent HPV [qHPV] vaccine. The routine cohort for vaccination is age 11–14 years (year 7 or 8 in secondary school depending on jurisdiction). The HPV vaccine is offered alongside other vaccines in the national school vaccination program (e.g. Diphtheria, Tetanus and Pertussis (DTaP) booster and varicella vaccines). In Australia, the National HPV Vaccination Program Register reports that for girls aged 14–15 years of age (as of mid-2012), 82 % received HPV dose 1, 78 % received HPV dose 2, and 71 % received HPV dose 3 (2013) [5].

### Strategies for school-based vaccination

In Australia, a school-based delivery is used, and parental consent is required for the vaccination to proceed. Schools distribute parental consent forms (with an information brochure) to all eligible students to take home for signing.^1^ However, students most often receive little, or no, education about HPV or HPV vaccination prior to immunisation [6, 7] and there is no requirement for separate consent/assent from the adolescent. Perhaps implicit is an expectation that parents will discuss the vaccine and share information with their adolescent. Where adolescents are absent on a school vaccination day, or have not provided a signed consent form, they can access vaccination through a local immunisation clinic or their general practitioner.

There is little evidence available to guide strategies for school-based vaccination. Our systematic review of practices for school-based vaccination implementation identified only one randomised controlled trial in 14 studies, which evaluated process [8, 9]. We identified the importance of well-designed vaccination education to promote understanding, and strategies to promote return of consent forms and to vaccinate students absent on school vaccination days [10–12]. In addition, our qualitative research in Sydney schools highlighted the importance of procedural issues, as well as knowledge and attitudes, to vaccination consent and completion [13–15].

### Adolescents’ lack of knowledge and understanding about the HPV vaccine in Australia

There is now clear evidence that many adolescents have little or no understanding of the vaccines they receive or the diseases they are intended to prevent. Our research in Australian schools has shown that, adolescents’ understanding, self-efficacy, and involvement in decision-making regarding HPV vaccination are low, and that their fear and anxiety are high [6, 14, 15]. Further, even after widespread media promotion, participation in the consent process and experiencing vaccination, there was still a low level of knowledge among adolescents in Australia [6, 16, 17]; and even internationally despite high vaccination rates [18, 19]. Despite media awareness campaigns, there is also some uncertainty about where adolescents can and should obtain reliable information about the vaccine [6, 20].

### Adolescent experience of needle related anxiety with vaccination

We have previously described significant adolescent anxiety associated with HPV vaccination in the school-based setting [15, 21] from adolescent, parent and nurse reports as well as vaccination-day observations, and international studies reveal similar and related concerns [18, 22]. We identified a range of strategies from our previous research, including: education of students about HPV and HPV vaccination prior to vaccination day; vaccinating adolescents in the morning so that they are not waiting all day; use of privacy screens during vaccination; bringing adolescents to the vaccination area in small groups to avoid extended waiting times; having a separate entrance and exit point so that vaccinated adolescents do not have contact with those still waiting; distraction techniques such as iPod use while waiting for vaccination, and relaxation techniques such as breathing exercises learned prior to vaccination day, which may reduce vaccination related anxiety [13, 15, 21].

### Parent and adolescent HPV vaccination decision making

We previously described HPV vaccination decision-making by parents and adolescents in a school setting [14]. We identified that parents face challenges discussing HPV and vaccination with their adolescents, and adolescents rarely participate in decision-making [14]. This situation is not optimal for several reasons, including: the missed opportunity for adolescents and parents to effectively communicate about important health issues and to support adolescents developing autonomy; there are important ethical reasons why adolescents should participate in consent processes regarding their own health [7, 21, 23]. For the school-based vaccination context, there are currently no tools to support an informed decision-making process that can be shared between parents and adolescents.

In this paper we describe the protocol of our cluster randomised controlled evaluation of decisional support and logistical strategies in school-based Human Papillomavirus (HPV) Vaccination of adolescents.

## Trial aims

The study aims to examine: 1) student knowledge about HPV vaccination; 2) psycho-social outcomes and 3) vaccination uptake.

## Trial methods

### Study design

Cluster randomised controlled trial occurring over two school years: 2013 and 2014.

### Sample

The overall target sample is 40 schools with year-8 enrolments above 100 students, stratified by Government, Catholic, and Independent sectors and geographical location, with up to 20 schools recruited in each of two states, Western Australia (WA) and South Australia (SA).

### Setting

The greater metropolitan area of Perth, WA, and Adelaide, SA.

### Participants

Male and female students in their first year of high school (year 8 in participating states). Key school personnel in participating schools and immunisation nurses.

### Advisory board

An Advisory Board exists that includes representatives from the government, Catholic and Independent education authorities and from health department and immunisation teams in both study jurisdications. The purpose of this Advisory Board is to advise on all aspects of the study. The board will meet regularly prior to study initiation and during the study with email communication throughout.

### Ethics and informed consent

We obtained ethical approval from the Department of Health WA Human Research Ethics Committee and Women’s and Children’s Hospital (WCHN) Human Research Ethics Committee. Approval was also obtained from the relevant WA and SA government authorities. We sought approval for a consent waiver from parents for the evaluation of this study [24]. In Australia, Human Research Ethics Committees may grant consent waivers when: involvement in the research carries no more than low risk; the benefits from the research outweigh the risk of harm from not seeking consent; participants would be expected to consent if asked; confidentiality is maintained; and attempts to obtain consent are very unlikely to lead to adequate response rates. These conditions all held for this research project and, accordingly, the project was granted ethical approval [25].

### Randomisation

#### Permuted block randomisation

Schools will be stratified by state (WA or SA) and sector (Government, Independent single-sex, Independent coeducational, Catholic single-sex, Catholic co-educational) and within strata, randomised using permuted blocks. Randomisation lists will be created using the software program Rand.exe [26]. The researchers recruiting schools and the contact people at the schools will be blind to which arm of the trial the school will be allocated to at the time the school agrees to take part in the study. The trial statistician, who will have no involvement in recruitment, will allocate schools using the randomisation list after the school has agreed to take part. Schools will be allocated according to the list in the order in which they are recruited. Where possible, schools will not be allocated until the target number in the strata is reached. After the school is allocated, recruiters and schools will no longer be blind to intervention/control status.

### Intervention

The intervention consists of three main components: 1) an adolescent intervention; 2) an HPV vaccine parent/adolescent decision support tool; and 3) logistical strategies. The adolescent intervention is designed to promote knowledge, decision-making involvement and confidence in vaccination (self-efficacy) and reduce vaccination-related anxiety in adolescents. The *decisional support tool* is designed for use by both adolescents and parents together to promote understanding and shared decision-making. Logistical strategies are designed to improve vaccination uptake, school vaccination processes and the adolescent experience. The intervention components are outlined in further detail below:

1. The *adolescent intervention* comprises: education taught through the school in an interactive lesson (see Additional file 1); a take-home magazine designed by and for adolescents; online components that can be accessed outside the school in a website and app for mobile devices; and distraction/relaxation methods to be used prior to and during vaccination, supported by nurses and teachers.
  Further information on the development of the adolescent intervention is detailed in a separate publication [27]. Briefly, education occurs via an 18 min animated film (on DVD) about HPV and HPV vaccination with seven chapters including: HPV- the virus; HPV vaccination; males and HPV; decision-making about HPV vaccination; what happens on the school vaccination day; cervical screening into the future. The film is designed to be screened in class, in an interactive way, with school personnel. A teacher user guide and supplement containing educational activities for each chapter accompany the DVD and other resources.
  The magazine includes a range of practical information about HPV and HPV vaccination, and the school vaccination day, in an appealing format. The magazine is designed to be taken home by students to read in their own time.
  A website (http://takechargehpv.org) and an app for mobile devices, including all educational resources will be available for students in intervention schools, to use in a flexible way. The website includes information about HPV and HPV vaccination and is intended to reinforce the information taught in class. Adolescents can re-watch the film clips, sign up to receive reminders about vaccination, and share their stories about being vaccinated.
  iPads will be available for use on vaccination day to assist with relaxation and distraction and will contain only the study app, which incorporates information from the website, the film chapters, the magazine, and relaxation exercises and distraction activities such as a painting tool.
2. A HPV vaccination *decisional support tool* (DST) designed for use by both adolescents and parents together in the home environment is being used in the study. It has been developed using input from parents and adolescents [28]. We have an evidence-based decision aid, developed according to internationally accepted standards for presenting balanced information about health care options [29]. This was tested and refined after input from male and female adolescent-parent dyads obtained in interviews [28]. This decision aid also incorporates innovative methods to facilitate discussion of sensitive topics and shared decision-making.
3. *Logistical strategies* will include methods for increasing consent form return such as direct mail-out of forms to parents (rather than being delivered to parents by adolescents); reminders (re-sending vaccination consent forms when they have not been returned); and non-material incentives for classroom consent form return (such as homeroom points) regardless of whether parental consent is granted. In addition, guidelines for nurses and teachers about the set-up of the vaccination room to minimise student anxiety, promote student privacy, and assist with the efficiency of vaccination processes (see Additional file 2), and distraction strategies to directly assist the management of adolescent anxiety will be implemented. In-school ‘mop-up’ vaccination for students who have a valid consent but were absent on a previous vaccination day will be provided both on standard vaccination days and through one additional visit per school after dose 3.

#### Training of staff to implement intervention

Prior to the commencement of the study in a school, study staff will offer training to school personnel in the educational and logistical components of the study. During this training a checklist of study activities will be provided to the coordinating staff member and discussed. A similar training session will also be completed with each of the school-based immunisation teams before the first vaccination day.

### Measure

#### HPV adolescent questionnaire (HAVIQ)

We have developed and validated a questionnaire (measure) to determine changes in adolescent knowledge, attitudes, fear and anxiety, self-efficacy, and decision-making [28]. The items in this measure have been informed by a review of existing questionnaires around HPV [30–34], the results of our own research [6, 7, 8, 13, 23], and an expert panel of academics working in related fields. The domains of the measure include: HPV and HPV vaccine knowledge and attitudes; HPV-related fear and anxiety ‘Feelings towards vaccination’; involvement in HPV vaccination decision-making; and HPV vaccination self-efficacy ‘Skills inventory’. Knowledge and attitudes, ‘Feelings towards vaccination’ and decision making involvement were measured with a series of questions (6, 6, and 8, respectively) each coded on a Likert scale (strongly agree to strongly disagree and scored 1–5); Skills inventory (5 questions) using a confidence scale from 0 to 100. The measure has been tested for face and content validity and internal consistency and test/re-test reliability. The questionnaire has been tested in six schools to determine preliminary impact on knowledge, vaccination self-efficacy and fear [35].

### Data collection

Psychosocial outcomes will be measured using the HPV Adolescent Vaccination Intervention Questionnaire (HAVIQ). School personnel will administer the entire HAVIQ questionnaire (all four domains) after the students have participated in the education intervention session, but before they have had dose 1 of the HPV vaccine in intervention schools, and prior to dose 1 in control schools. Both groups of schools will complete the ‘Skills inventory’ and ‘Feelings towards vaccination’ components of the questionnaire before dose 2 of the HPV vaccine to measure the change in these domains after personal experience with vaccination. The ‘Knowledge’ component of the questionnaire will be given again prior to dose 3 of the HPV vaccine to measure knowledge retention over time. Self-reported vaccination status will be included in the questionnaire prior to dose 3.

#### Vaccination uptake

We will obtain de-identified school-level immunisation uptake data from each State’s Health Department. De-identified student vaccination data will be linked to questionnaire data via codes generated by participating schools’ administration.

#### Time taken to immunise

This will be documented by the school-based immunisation nurses on the study “School-based Immunisation Log for Nursing Staff” on each vaccination day. The start and end time will be recorded, as well as the total number of hours/minutes spent vaccinating the students, excluding any breaks.

#### Standard procedures for vaccination in all schools

Consent for vaccination forms will be sent out as per normal operating procedures described in the relevant state guidelines. *Vaccination Room set*-*up*: Room set up to follow normal operating procedures described in relevant state guidelines. *Mop*-*ups*: To follow normal operating procedures described in relevant state guidelines.

### Statistical methods/analysis

#### Power calculation

To allow for the possibility of schools dropping out of the study, we increased the number of schools by 10 % and aimed to recruit a total of 40 schools. A total of 36 schools allows for the detection of a change in the percentage of students vaccinated from 70 to 80 % at a significance level of 0.05 and with a power of 80 % and assuming an intraclass correlation coefficient (ICC) of 0.05 [36]. We estimated the ICC from a pilot study in NSW to be 0.04, and other cluster trials of adolescent health behaviours have reported ICC’s of less than 0.05 [37]. We estimated, that on average, each school of the 36 schools would have 150 students giving a total final sample size of 5400 students.

With a total of 36 schools, we will be able to detect the following differences in means between the intervention and control groups for the four secondary outcomes: 1) Knowledge–minimum detectable difference “equals” = 0.7; 2) Fear/anxiety–minimum detectable difference “equals” = 0.7; 3) Decision making–minimum detectable difference “equals” = 0.5; 4) Skills inventory–minimum detectable difference “equals” = 18; all assuming an ICC of 0.05, power of 80 %, significance of 0.05 and standard deviations derived from a pilot study evaluating each of these measures and allowing for a 40 % non-completion of these outcomes [36, 37].

#### Primary outcome analysis

The primary analysis will compare vaccination rates using the Mantel-Haenszel method, taking into account the stratification by year, state and school sector and adjusted for clustering [36]. In addition, logistic regression models will be used to adjust for baseline vaccination rates (average of the previous 2 years), school type (single-sex, or mixed), school size and SEIFA index using generalized estimating equations (GEE) with robust standard errors. The level of implementation of the intervention will also be included.

#### Secondary outcome analysis

Mean change in scores of knowledge, self-efficacy, fear/anxiety, and decision-making will be compared between groups using two-sample t-tests with appropriate adjustment for clustering. Baseline scores for knowledge will be taken from the control group. Proportion of consent form returns will be compared between groups prior to dose 1 using Chi-square test with appropriate adjustment for clustering. Time taken to vaccinate will be measured by nurses during each immunisation day in both intervention and control schools. It will be calculated as the mean time to vaccinate 50 students in intervention schools compared with control schools. Mean time to vaccinate will be compared between groups in both years of the study to determine effect of the intervention over time. We will investigate whether the intervention improves vaccination rates through its effect on fear reduction, and self-efficacy increase, by comparing estimates of the effect of the intervention both with and without adjustment for the possible intermediary variables of fear/anxiety, and self-efficacy. These logistic regression models will use GEE and robust standard errors to account for the clustering.

#### Process evaluation

1. Implementation of intervention
  *Teachers and nurses logs*: During the intervention, school personnel and immunisation nurses will complete study logs. The first log will document the implementation of the education intervention to be completed by school personnel, the second log will document vaccination day processes to be completed by immunization nurses, and the third log will document logistics implemented and observed by supervising school personnel on vaccination day.
2. Qualitative evaluation
  We will undertake this in a sub-sample of intervention and control schools, which we term the ‘case-study schools’. Data collection will include observations of vaccination day, semi-structured interviews with teachers, school nurses, immunisation nurses and parents, and semi-structured focus groups with students to provide a more in-depth understanding of the mechanisms for change promoted by the intervention. The interviews and focus groups will be conducted primarily to explore the effects of the study intervention and what aspects of the intervention are most useful and what aspects are less useful. Qualitative ethnographic methods have been used with success to evaluate previous similar investigations [6, 14].
  Six intervention schools and 6 control schools will serve as case studies during the study. Case studies are detailed analyses of persons, events, decisions, projects, policies, institutions, programs, interventions or other systems that are studied holistically [38]. Case studies can draw on qualitative and quantitative data to provide in-depth examples of schools as sites in which knowledge, perceptions and experiences of HPV and HPV vaccination are effectively contextualised. This method will allow better explanation of the effects of the intervention by comparing and contrasting case studies.
  We aim to recruit 6–10 students for at least one focus group and 6–8 parents for one focus group or at least 4 individual interviews in each case study school. We will interview at least one school personnel member and at least one immunisation nurse for each selected school. If data collected from one school is insufficient for data saturation, then an additional school may be recruited.
  All qualitative data will be digitally recorded, and transcribed verbatim. The qualitative evaluation aims to explore in depth possible mechanisms for any observed effect of the intervention on primary and secondary outcomes. We will analyse the qualitative data using thematic and discourse analysis [39–42].

## Discussion

This study is unique for several reasons. The intervention is multi-faceted and situated in the community, which facilitates direct translation to the real world. Rigorous evaluation methods are used. A special strength of this study is the use of qualitative data collection to provide in-depth understandings of mechanisms of change. Adolescents will be informed participants in vaccination. Further, this study will evaluate a novel tool to promote adolescent and parental cooperation in vaccination decision-making. The intervention may impact on uptake of other vaccines offered in the school program, as aspects of the intervention are generic for vaccines (such as consent form processes, mop up vaccination, and tools to promote self-efficacy and reduce anxiety); educational resources could also be extended to include the other vaccines offered, to maximize public health benefit.

There is international interest in how to vaccinate the young adolescent age group, as many countries are experiencing challenges in achieving HPV vaccination coverage targets. Adolescents are outside the age range of most established childhood vaccination programs. If this project demonstrates an increase in adolescent knowledge and self-efficacy, improves decision involvement, and reduces vaccine related anxiety, the findings of this study will be of interest not only to the Australian school vaccination program, but all countries.

### Confidentiality of named data

The Principal Investigator or delegate will maintain source documentation for every participant enrolled in the study and all study forms for each enrolled participant will be stored securely in locked cabinets and will only be accessed by appropriately trained research staff. Any publications based on the data will only provide the allocated pseudonym of the participant to protect their identity.

This will be the first study of its kind to provide an evidence-based for successful school based vaccination against HPV. This study should provide data on what interventions support student knowledge and understanding of HPV and vaccination, improve their involvement in consent and decision-making (supporting their future health autonomy), and improve their experience of vaccination. Building on the complexity of the intervention, we should be able to determine which logistical strategies improve vaccination uptake in school-based vaccination. These data will provide essential evidence going forward to support the use of school based platforms for delivery of vaccination to adolescents. It will also provide detailed guidance to other countries that are considering utilising schools to offer vaccination to adolescents for the first time. There is huge international interest in how to vaccinate the young adolescent age group given the enormous potential for future health benefit. This is a challenge for many countries as adolescents are outside the age range of most established childhood vaccination programs; without appropriate vaccination delivery systems in place, such as effective school based vaccination, uptake will not come close to the desired targets.

## Additional files

Additional file 1:
**HPV.edu: An educational intervention about HPV and the HPV vaccine.** (DOC 50 kb) Additional file 2:
**Vaccination logistical interventions.** (DOC 145 kb)

## Footnotes

1 In each state and territory of Australia, parents are supplied with a fact sheet with information about all the vaccines offered through the national school based vaccination program.

## References

[1] England PH. Annual HPV vaccine coverage 2013 to 2014: by PCT, local authority and area team. Public Health England 2014, [cited 2015]; Available from: https://www.gov.uk/government/statistics/annual-hpv-vaccine-coverage-2013-to-2014-by-pct-local-authority-and-area-team.

[2] Elam-Evans LD, Yankey D, Jeyarajah J, Singleton JA, Curtis RC, MacNeil J (2014). National, regional, state, and selected local area vaccination coverage among adolescents aged 13–17 years—United States, 2013. Morbidity and Mortality Weekly Report (MMWR).

[3] Smith MA, Canfell K, Brotherton JM, Lew JB, Barnabas RV (2008). The predicted impact of vaccination on human papillomavirus infections in Australia. Int J Cancer.

[4] Marshall HS, Collins J, Sullivan T, Tooher R, O'Keefe M, Skinner SR (2013). Parental and societal support for adolescent immunization through school based immunization programs. Vaccine.

[5] National HPV Vaccination Program Register (2015). Coverage data.

[6] Cooper Robbins SC, Bernard D, McCaffery K, Brotherton J, Garland S, Skinner SR (2010). “Is cancer contagious?”: Australian adolescent girls and their parents: making the most of limited information about HPV and HPV vaccination. Vaccine.

[7] Robbins SC, Bernard D, McCaffery K, Brotherton JM, Skinner SR (2011). “I just signed”: factors influencing decision-making for school-based HPV vaccination of adolescent girls. Health Psychol.

[8] Cooper Robbins S, Ward K, Skinner SR (2011). School-based vaccination: a systematic review of process evaluations. Vaccine.

[9] Skinner SR, Imberger A, Nolan T, Lester R, Glover S, Bowes G (2000). Randomised controlled trial of an educational strategy to increase school-based adolescent hepatitis B vaccination. Aust N Z J Public Health.

[10] Rand CM, Szilagyi PG, Albertin C, Auinger P (2007). Additional health care visits needed among adolescents for human papillomavirus vaccine delivery within medical homes: a national study. Pediatrics.

[11] Brown ECF, Little P, Leydon GM (2010). Communication challenges of HPV vaccination. Fam Pract.

[12] Kessels SJ, Marshall HS, Watson M, Braunack-Mayer AJ, Reuzel R, Tooher RL (2012). Factors associated with HPV vaccine uptake in teenage girls: a systematic review. Vaccine.

[13] Robbins SC, Bernard D, McCaffery K, Skinner SR (2010). ‘It’s a logistical nightmare!’ Recommendations for optimising human papillomavirus school-based vaccination experiences. Sex Health.

[14] Robbins SC, Bernard D, McCaffery K, Brotherton JM, Skinner SR (2010). “I just signed”: factors influencing decision-making for school-based HPV vaccination of adolescent girls. Psychology.

[15] Bernard DM, Cooper Robbins SC, McCaffery KJ, Scott CM, Skinner SR (2011). The domino effect: adolescent girls’ response to human papillomavirus vaccination. The Medical Journal of Australia.

[16] Mitchell A, Patrick K, Heywood W, Blackman P, Pitts M, National Survey of Australian Secondary Students and Sexual Health (2014). National survey of Australian secondary students and sexual health 2013.

[17] Burns K, Davies C, Wright K, McLeod J (2015). Constructions of young women’s health and wellbeing in neoliberal times: a case study of the HPV Vaccination Program in Australia. Rethinking youth wellbeing: critical perspectives.

[18] Kennedy C, Brunton CG, Hogg R (2014). ‘Just that little Bit of Doubt’: Scottish Parents’, teenage Girls’ and health professionals’ views of the MMR, H1N1 and HPV vaccines. International Journal of Behavioural Medicine.

[19] Hilton S, Smith E (2011). “I thought cancer was one of those random things. I didn’t know cancer could be caught…”: adolescent girls’ understandings and experiences of the HPV programme in the UK. Vaccine.

[20] Davies C, Burns K (2014). Mediating healthy citizenship in the HPV vaccination campaigns. Feminist Media Studies.

[21] Braunack-Mayer A, Skinner SR, Collins J, Tooher R, Proeve C, O'Keefe M (2015). Ethical challenges in school-based immunisation programs for adolescents: a qualitative study. Am J Public Health.

[22] Henderson L, Clements A, Damery S, Wilkinson C, Austoker J, Wilson S (2011). ‘A false sense of security’? understanding the role of the HPV vaccine on future cervical screening behaviour: a qualitative study of UK parents and girls of vaccination age. J Med Screen.

[23] Skinner SR, Cooper Robbins SC (2010). Voluntary school-based human papillomavirus vaccination: an efficient and acceptable model for achieving high vaccine coverage in adolescents. J Adolesc Health.

[24] Esbensen FA, Melde C, Taylor TJ, Peterson D (2008). Active parental consent in school-based research How much is enough and how do we get it?. Eval Rev.

[25] NHMRC. Chapter 2.3: Qualifying or waiving conditions for consent; National Statement on Ethical Conduct in Human Research. 2007 2015 [cited 2015 1st May 2015]; Available from: https://www.nhmrc.gov.au/book/national-statement-ethical-conduct-human-research-2007-updated-december-2013/chapter-2-3-qualif.

[26] Piantadosi S (2005). Clinical trials: a methodologic perspective.

[27] Cooper SC, Davies C, Mahendran K, Blades, J, Stoney T, Marshall H, et al. Development of an HPV Vaccination Intervention for Australian adolescents*.* Health Education Journal, in press.

[28] Cooper S, Davies C, Fernández-Cerdeño A, Cree RA, Fletcher H, McCaffery K, et al. Creation of a decision aid for shared HPV vaccination decision-making between parents and adolescents. In review.

[29] Elwyn G, O’Connor A, Stacey D, Volk R, Edwards A, Coulter A (2006). Developing a quality criteria framework for patient decision aids: online international Delphi consensus pro. BMJ.

[30] Kahn JA, Rosenthal SL, Jin Y, Huang B, Namakydoust A, Zimet GD (2008). Rates of human papillomavirus vaccination, attitudes about vaccination, and human papillomavirus prevalence in young women. Obstet Gynecol.

[31] Kahn JA, Rosenthal SL, Hamann T, Bernstein DI (2003). Attitudes about human papillomavirus vaccine in young women. Int J STD AIDS.

[32] Marlow LA, Waller J, Evans RE, Wardle J (2009). Predictors of interest in HPV vaccination: a study of British adolescents. Vaccine.

[33] Forster AS, Marlow LA, Wardle J, Stephenson J, Waller J (2010). Understanding adolescents’ intentions to have the HPV vaccine. Vaccine.

[34] Lloyd GP, Marlow LA, Waller J, Miles A, Wardle J (2009). An experimental investigation of the emotional and motivational impact of HPV information in adolescents. J Adolesc Health.

[35] McBride K, et al. Evaluating an educational intervention for school-based HPV vaccination: development and validation of scale. In review.

[36] Donner A, Klar N (2000). Design and analysis of cluster randomization trials in health research.

[37] Murray DM, Blistein JL (2003). Methods to reduce the impact of intraclass correlation in group-randomized trials. Eval Rev.

[38] Yin RK (2014). Case study research : design and methods.

[39] Lupton D (2012). Medicine as culture : illness, disease and the body.

[40] Lupton D (1992). Discourse analysis: a new methodology for understanding the idealogies of health and illness. Aust J Public Health.

[41] Gillen J, Petersen A, Somekh B, Lewin C (2005). Discourse analysis. Research methods in the social sciences.

[42] Braun V, Clarke V (2006). Using thematic analysis in psychology. Qual Res Psychol.

