# Supplementary material for: HPV.edu study protocol: a cluster randomised controlled evaluation of education, decisional support and logistical strategies in school-based human papillomavirus (HPV) vaccination of adolescents
Source: BMC Public Health. 2015 Sep 15;15:896. doi: 10.1186/s12889-015-2168-5 (PMC4572679; doi:10.1186/s12889-015-2168-5)
Supplement: Additional file 1: — HPV.edu: An educational intervention about HPV and the HPV vaccine. (DOC 50 kb) [file 12889_2015_2168_MOESM1_ESM.doc]

**Additional file 1**

5.3.2 LOGISTICAL INTERVENTIONS

Prior to the commencement of the study in a school, study staff are required to train the nurses/teachers in the logistical components of the study. During this training a checklist is to be provided to the co-ordinating staff member and discussed. The checklist details every aspect of the study intervention and is specific to whether the school has been allocated to the intervention or control arm of the study. The checklist assists the research team in ensuring that all aspects of the study have been addressed with the school staff including the completion of the related study documents (eg supervision log and if applicable the teacher log and supervision consent forms)

A similar training session will also be completed with each of the school-based immunisation teams before the first vaccination day. The checklist should be completed by research staff to ensure that all components of the intervention have been addressed.

5.3.2.1 Intervention Schools

*Consent Return*

HPV.edu aims to get high consent form return, regardless of consent to vaccinate which is in accordance with state school based immunisation guidelines. Prior to the government immunisation consent forms going home to students’ parents, research staff will add the ‘HPV summary booklet/ HPV Shared Decision-making booklet’ to the vaccination consent package where possible. If appropriate and possible, parental consent forms for the HAVIQ will also be added to these government vaccination consent packs. Research staff will document this process on the “HPV.edu study check-list” and any deviations will be noted.

*Vaccination Room set-up*

Researchers will meet with school personnel about room set-up to maximise privacy and vaccination flow (how to bring small groups down—use peer leaders or teachers) and how to do logs.

Research staff will liaise with immunisation teams that vaccinate in HPV.edu study schools so they are aware that schools are encouraged to follow these guidelines.

Currently Australian states have comprehensive guidelines regarding vaccination room set-up. In intervention schools, research staff will work with immunisation teams to encourage and assist in the adherence to their own guidelines as far as they overlap with the study logistical strategy guidelines. For example, at the time of the study initiation, one Australian state’s school based vaccination guidelines included the following:

- Ideally the immunisation room should be large enough for 2 or 4 nurses working in pairs, the 1 or 2 students being vaccinated and an observation area for students post vaccination.
- It works best if there is a separate entry and exit door:
  - students are required to wait their turn outside the entrance door (consider weather conditions)
  - an observation area is required near the nurses for students to remain for 15 minutes before returning to their classroom (in case of fainting or a reaction)

Waiting area for students **IN**

Nurse’s
table

before immunisation

Admin
table

Nurse’s
table

**OUT** Quiet

observation

area

- Additional requirements include equipment such as privacy screens.
- Small groups consist of classroom sizes, up to 30 students and school personnel are utilised for crowd control and supervision pre and post vaccination.

*HPV.edu iPads*

Immunisation Teams will be provided with the HPV.edu app on iPad. The ipads are to be used for education and distraction purposes for adolescents either while they are waiting in line or during the vaccination procedure. This will be organised at the discretion of the immunisation teams.

All iPads must be labelled and the serial numbers and iPad number needs to be recorded, as well as which immunisation team has what iPads, in a log by research staff so that they can be tracked if any problems are encountered.

Research staff will provide iPad training to the school-based immunisation nurses and encourage them to direct students to distraction and education components of the app prior to vaccination. Where school personnel have also received adequate iPad training they too may assist with this task.

Schools and nurses will need to complete their specific study logs with fidelity checklists regarding its use.

Other studies resources are available at: <http://takechargehpv.org>

*In school ‘mop-ups’*

Mop-ups (in school catch-up visits) are encouraged in some state immunization guidelines. Research staff will encourage all school based immunisation teams to perform at least one mop-up visit in all intervention schools, reimbursement of time may be offered, where permissible. The timing of these visits will be at the discretion of the individual immunisation teams. The immunisation nurses will be asked to complete the “School-based Immunisation log for nursing staff” for each mop up visit. Immunisation teams will continue with their usual practice in control schools and immunisation logs will also be completed these schools, to capture any ‘mop up visits and doses given’.

It is common practice for parents/guardians of students who miss vaccination doses at school to receive notification from immunisation teams about free vaccination clinics to receive missed vaccinations. This process will continue for all schools participating in the study.

*Time taken to immunise*

This will be documented by the school-based immunisation nurses on the HPV.edu “School-based Immunisation Log for Nursing Staff” on each vaccination day. Ideally the start and end time should be recorded, as well as the total number of hours/minutes spent vaccinating the students, excluding any breaks.

*Vaccination up-take*

The state health authority will provide de-identified student vaccination data using the codes generated by the school’s administration to allow linking to student questionnaire data..

**5.3.2.2 Logistical Intervention in Control Schools**

*Consent Return*

Consent forms sent out as per normal operating procedures described in the relevant state guidelines.

*Vaccination Room set-up*

Room set up to follow normal operating procedures described relevant state guidelines.

*Mop-ups*

Mop-ups are to follow normal operating procedures described relevant state guidelines.

*Time taken to immunise*

This will be documented by the school-based immunisation nurses on the HPV.edu “School-based Immunisation Log for Nursing Staff” on each vaccination day. Ideally the start and end time should be recorded, as well as the total number of hours/minutes spent vaccinating the students, excluding any breaks.

*Vaccination up-take*

The state health authority will provide de-identified vaccination data using the codes generated by the school’s administration as used on the student questionnaire.
