# Supplementary material for: HPV.edu study protocol: a cluster randomised controlled evaluation of education, decisional support and logistical strategies in school-based human papillomavirus (HPV) vaccination of adolescents
Source: BMC Public Health. 2015 Sep 15;15:896. doi: 10.1186/s12889-015-2168-5 (PMC4572679; doi:10.1186/s12889-015-2168-5)
Supplement: Additional file 2: — Vaccination logistical interventions. (DOC 145 kb) [file 12889_2015_2168_MOESM2_ESM.doc]

**Additional file 2**

HPV.edu

An educational intervention about HPV and the HPV vaccine

These educational resources have been developed by researchers from the University of Sydney, the University of Adelaide, the Women's and Children's Hospital, and the Telethon Institute for Child Health Research. The development of these resources was made possible through research grant funds from GlaxoSmithKline Australia and the National Health and Medical Research Council.

Teachers guide for using HPV.edu intervention resources [24](#__RefHeading___Toc300316429)

What are the resources for the HPV educational intervention? [24](#__RefHeading___Toc300316430)

Educational Resources [24](#__RefHeading___Toc300316431)

How do I use these HPV education resources? [24](#__RefHeading___Toc300316432)

RESOURCE 1: HPV.EDU FILM CHAPTERS [24](#__RefHeading___Toc300316433)

RESOURCE 2: TEACHER SUPPLEMENT [24](#__RefHeading___Toc300316434)

RESOURCE 3: MAGAZINE—TAKE-HOME INFORMATION DESIGNED WITH AND FOR ADOLESCENTS [25](#__RefHeading___Toc300316435)

RESOURCE 4: WEBSITE [25](#__RefHeading___Toc300316436)

RESOURCE 5: DISTRACTION/RELAXATION APP [25](#__RefHeading___Toc300316437)

RESOURCE 6: HPV ADOLESCENT QUESTIONNAIRE (HAVIQ) [25](#__RefHeading___Toc300316438)

REPEATING COMPONENTS OF THE QUESTIONNAIRE [26](#__RefHeading___Toc300316439)

RESOURCE 7: DECISION AID [26](#__RefHeading___Toc300316440)

Why were these HPV education resources developed? [26](#__RefHeading___Toc300316441)

How will the HPV.edu research team know what resources I have used, or how much lesson time I have taken to teach students using the educational resources? [27](#__RefHeading___Toc300316442)

What is the relationship between these resources and the curriculum in my State? [27](#__RefHeading___Toc300316443)

HPV.edu and the Western Australian Curriculum: Health and Physical Education Learning Area Statement [27](#__RefHeading___Toc300316444)

Teacher Log [29](#__RefHeading___Toc300316445)

Materials and description [32](#__RefHeading___Toc300316446)

How to use these curriculum support materials [32](#__RefHeading___Toc300316447)

Contents [32](#__RefHeading___Toc300316448)

PART 1: ABOUT HPV TEACHER SUPPLEMENT [33](#__RefHeading___Toc300316449)

Activity 1: HPV Question Panel (14 minutes) [33](#__RefHeading___Toc300316450)

Activity 2: Crossword Puzzle (6 minutes) [35](#__RefHeading___Toc300316451)

PART 2: HPV VACCINATION TEACHER SUPPLEMENT [37](#__RefHeading___Toc300316452)

Activity 3: Matching Game (5 minutes) [37](#__RefHeading___Toc300316453)

Activity 4: Educate the Public (30 minutes) [38](#__RefHeading___Toc300316454)

PART 3: DECISION-MAKING [39](#__RefHeading___Toc300316455)

TEACHER SUPPLEMENT [39](#__RefHeading___Toc300316456)

Activity 5: Talk with your Parent (12 minutes) [39](#__RefHeading___Toc300316457)

Activity 6: The Decision-Making Process (10 minutes) [41](#__RefHeading___Toc300316458)

PART 4: PRACTICAL TEACHER SUPPLEMENT [43](#__RefHeading___Toc300316459)

Activity 7: Meditation Exercise (10 minutes) [43](#__RefHeading___Toc300316460)

PART 5: FUTURE TEACHER SUPPLEMENT [44](#__RefHeading___Toc300316461)

Activity 8: Summary (12 minutes) [44](#__RefHeading___Toc300316462)

Activity 9: HPV Bingo (20 minutes) [46](#__RefHeading___Toc300316463)

**Teachers guide for using HPV.edu intervention resources**

**What are the resources for the HPV educational intervention?**

The HPV educational intervention contains 6 resources. Resources 1 and 2 are to be delivered by teachers with students in a classroom setting; resources 3 and 4 are to be used by students in their own time. Resource 5 is to be used by the student while s/he is waiting to receive the HPV vaccination (during each of the 3 doses). Resource 6, will be sent home with the student as part of the HPV information and consent package, and is to be used by a parent/guardian with their adolescent. Resource 7 is the HPV Student Questionnaire, an assessment tool measuring knowledge and skills gained from the resources, to be given to students after they have accessed the other resources and just before they receive the 1^st^ dose.

**Educational Resources**

1. 7 HPV.edu film chapters
2. Teacher Supplement to film chapters
3. Magazine (to go home with students after HPV education)
4. Website (for students to access outside of class time)
5. HPV.edu app (to use on iPod touch or iPad while waiting to be vaccinated)
6. HPV Student Questionnaire (HAVIQ)
7. Decision Aid (to be used by the student with a parent or guardian)

**How do I use these HPV education resources?**

**RESOURCE 1: HPV.EDU FILM CHAPTERS**

The HPV.edu film chapters are to be screened in class (such as a Health and Physical Education class, or homeroom) by a teacher, and used with the Teacher Supplement, which contains educational activities to undertake with students. The film chapter content covers: HPV1: What is HPV?; HPV2: What is HPV vaccination?; HPV3: Boys and HPV; HPV4: HPV Decision-Making; HPV5: Vaccination on the day; HPV6: Vaccination in the future; HPV7: Recap; HPV8: Credits. After each HPV.edu film chapter is screened for students, you can field questions from students and/or consult the Teacher Supplement (Resource 2), which contains educational activities designed to assist students with their learning about HPV and HPV vaccination.

The HPV.edu film chapters are available at: <http://takechargehpv.org/>

**RESOURCE 2: TEACHER SUPPLEMENT**

The Teacher Supplement outlines curriculum content to be used by teachers with students in between the HPV.edu film chapters. The purpose of these mini lesson plans is to allow students to think about what they have learned from the HPV and vaccination video segments. Through the interactive activities, students will have a better understanding of concepts presented in the videos, including why they should have the vaccine; what are the benefits and limitations of the vaccine; exactly what happens when they get vaccinated as well as the science behind the vaccine. As they become better informed, this will help promote confidence and self-efficacy with vaccination allowing for a positive experience for students, staff, and nurses on vaccination day.

**RESOURCE 3: MAGAZINE—TAKE-HOME INFORMATION DESIGNED WITH AND FOR ADOLESCENTS**

You will be issued with magazines focusing on HPV and HPV vaccination. Please hand one magazine to each student at the end of the same class in which the film chapters and teacher facilitated activities in the Teacher Supplement will take place. Students should be instructed to read the magazine in their own time.

**RESOURCE 4: WEBSITE**

The website is designed to be accessed by students outside of school. The website provides adolescents with information about HPV and HPV vaccination.  It is intended to reinforce the information taught in class: adolescents can re-watch the film clips and access information about HPV and HPV vaccination.

Please make students aware of the website during the lesson in which the film chapters are screened and the website address will be printed in the magazine.

The website is available at: http://takechargehpv.org/

**RESOURCE 5: DISTRACTION/RELAXATION APP**

This application (or “app”) will be available for adolescents via iPod touch and/or iPad on the day of vaccination. The app will bring together information from the website, including the films and relaxation exercises, making this content available without requiring an Internet connection. Students waiting in a queue to be vaccinated will have access to the app, which will act as a distraction tool.

**RESOURCE 6: HPV ADOLESCENT QUESTIONNAIRE (HAVIQ)**

The questionnaire measures student knowledge, self-efficacy, decision involvement and fear/ anxiety. The questionnaire is an assessment tool that has been developed using rigorous methods and is thus accurate at assessing abilities in each of the domains. It is to be used by teachers for their own assessment of student learning about HPV as well as for use by researchers to determine how well the educational resources work.

Teachers will administer the questionnaire, **after** the education resources have been given to the students and **before** the first HPV vaccination.

**REPEATING COMPONENTS OF THE QUESTIONNAIRE**

- The ‘Skills inventory’ and ‘Feelings towards vaccination’ components of the questionnaire are also given before dose 2 of the HPV vaccine.
- The ‘Knowledge’ component of the questionnaire is given again pre dose 3 of the HPV vaccine.

**RESOURCE 7: DECISION AID**

The *Decision Aid* is designed for use by both adolescents and parents together, in the home environment. It will be available via web (on an app or a browser) and in hard copy. The hard copy version of the decision aid will be sent home with the student along with consent forms prior to the first dose of vaccination. The decision aid is designed to help parents and adolescents make an informed decision when consenting to the HPV vaccine; the decision aid presents facts only in an unbiased way.

**Why were these HPV education resources developed?**

Educational HPV and HPV vaccination interventions have been developed for adolescent girls and boys. The purpose of these educational interventions is to increase adolescent knowledge and understanding and to increase confidence and self -efficacy with vaccination. These educational resources are designed to align with Health and Physical Education curriculum frameworks and to be implemented at a school level, with minimal impact on school and teacher resources.

Low levels of knowledge mean that students don’t understand the benefits and limitations of vaccination, whether vaccination is in their best interest and what it means for their future health. Low knowledge and understanding can also exacerbate vaccination anxiety, and our previous research showed vaccination time increased with increasing student anxiety. Student anxiety is unpleasant for adolescents, as well as those administering the vaccine and the teachers who look after the students.

These educational resources will allow students to become informed and confident in their HPV vaccination decision. Increasing access to HPV and HPV vaccination information will empower adolescents and their parents make a health-related decision based on fact instead of misconceptions and opinions. The purpose of these educational resources is to increase HPV and HPV vaccination understanding, to provide information for future health, and to allow the school-based vaccination process to run more effectively. These aims are in line with best practice policy for vaccination in general, as well as in line with Health and Physical Education curriculum/s and associated syllabi.

**How will the HPV.edu research team know what resources I have used, or how much lesson time I have taken to teach students using the educational resources?**

You will be required to keep a log of the resources used and time spent teaching from these resources. The log is attached on a separate sheet with this user guide. You need to fill out this log after you have undertaken the HPV.edu education sessions/s with students. Please submit the log to the nominated HPV.edu project liaison person at your school, so that the research team can collect these documents.

**What is the relationship between these resources and the curriculum in my State?**

The resources have been designed to meet the curriculum guide and outcomes for Physical Health and Education in South Australia and Western Australia. The HPV.edu research team has also consulted educational professionals from The Australian Curriculum, Assessment and Reporting Authority (ACARA) to ensure that the resources will also be consistent with the outcomes of the National Curriculum.

**HPV.edu and the Western Australian Curriculum: Health and Physical Education Learning Area Statement**

The HPV.edu resources address the ‘Knowledge and Understandings’, and ‘Self-management Skills’ phases of curriculum development. Acquiring knowledge and understanding HPV and HPV vaccination is relevant to the following phases of development identified in the curriculum:

| **The scope of the curriculum—phases of development. Early Adolescence: years 7-10** |  |
| --- | --- |
| Knowledge and Understandings | They should be provided with opportunities to discuss the physical, mental, emotional and social changes associated with growth and development. They need to acquire  knowledge about the processes of  conception, pregnancy, childbirth and  ageing.  In early adolescence, students need to  develop strategies for reducing risks  associated with certain lifestyle behaviours.  Teachers should provide advice about how to avoid lifestyle diseases, including heart disease and communicable diseases (e.g. HIV/AIDS, sexually-transmitted diseases and hepatitis).  They [students] need to be able to view the media critically and to carefully analyse the messages and information which are conveyed in relation to nutrition, drugs, sexual activity and relationships. |
| Self-management Skills | Students are encouraged to examine and  apply their self-management skills to the  planning of short-term and longer-term  health and physical activity goals. With  practice they can effectively utilise these  skills to cope with changing and challenging situations and to provide support for others.  Students should apply the decision-making process in a variety of familiar and unfamiliar situations. They should be able to analyse the processes and outcomes of decision-making, taking into account their values and those of others (e.g. the media).  Students are assisted in developing decision making skills through role-play situations such as justifying and informing others of their choices (e.g. choosing non-alcoholic drinks at peers’ parties), discussing drug issues with parents or refereeing a sport.  Through class activities such as these,  students can empathise with and consider the views of others in their own decision  making. |

**Teacher Log**

**Date:**

**1) Lesson type(s) (e.g. Physical Education and Health/Homeroom):**

**2) Year of students (year 8 or 9):**

**3). Number of students included in lesson (all students, most, some, few)**

**4) Sex of students (male only, female only, coeducational):**

**5) Lesson time spent teaching (please include how many sessions and how many minutes each session included teaching HPV.edu):**

**6) Resources used (please indicate below which resources you did and did not use by placing a tick in the relevant boxes below):**

| **Name of resource** | **Used** | **Not Used** |
| --- | --- | --- |
| **Film chapter HPV1: What is HPV?** |  |  |
| **Film chapter HPV2: What is HPV Vaccination?** |  |  |
| **Film chapter HPV3: Boys and HPV** |  |  |
| **Film chapter HPV4: HPV Decision-Making** |  |  |
| **Film chapter HPV5: Vaccination on the day** |  |  |
| **Film chapter HPV6: Vaccination in the Future** |  |  |
| **Film chapter HPV7: Credits** |  |  |
| **Teacher Supplement Activity 1:**  **HPV Question Panel** |  |  |
| **Teacher Supplement Activity 2:**  **Crossword Puzzle** |  |  |
| **Teacher Supplement Activity 3:**  **Matching Game** |  |  |
| **Teacher Supplement**  **Activity 4: Educate the Public** |  |  |
| **Teacher Supplement**  **Activity 5: Talk with your Parent** |  |  |
| **Teacher Supplement**  **Activity 6: The Decision-Making Process** |  |  |
| **Teacher Supplement**  **Activity 7: Meditation Exercise** |  |  |
| **Teacher Supplement**  **Activity 8: Summary** |  |  |
| **Teacher Supplement**  **Activity 9: HPV Bingo** |  |  |
| **HPV Student Questionnaire**  **(HAVIQ)** |  |  |
| **Magazine (compulsory for students to receive this in class and read it at home).** |  |  |
| **HPV.edu website**  **(compulsory for students to receive information about the website in class and read it at home).** |  |  |

**7) Please indicate the number of students that received the magazine;**

| **Magazine** | **Number of students that received the magazine** | **Number of students that did not receive the magazine** |
| --- | --- | --- |
|  |  |  |

**8) Please indicate the number of students undertook the HPV Student Questionnaire:**

| **HPV Student Questionnaire**  **(HAVIQ)** | **Number of students that DID undertake the questionnaire** | **Number of students that DID NOT undertake the questionnaire** |
| --- | --- | --- |
|  |  |  |

**9) What were some of the questions asked by students about HPV or HPV vaccination?**

**10) Were you able to answer all of these questions with the resources and training made available to you through this intervention? Why/Why not?**

**11) Do you have any comments or suggestions to make regarding the resources?**

**HPV.edu Teacher Supplement**

The following document outlines curriculum content to be used by teachers with students between the films. Please note that the resources required for these activities are contained at the end of this folder.

**Materials and description**

The purpose of these mini lesson plans is to allow students to think about what they have learned from the HPV and vaccination video segments. Through the following interactive activities, students will have a better understanding of concepts presented in the videos, and will be able to understand the science behind the HPV vaccine they are offered in year 8. This will help alleviate fear and lack of knowledge allowing for a positive experience for students, teachers, and nurses on vaccination day.

**How to use these curriculum support materials**

These materials are designed to support a full 50-60 minute lesson, but you can also elect to select individual segments based on your time constraints or for integration with other content or activities. We strongly recommend doing at least one activity in segment 3 (either activity 5 or 6). Below are some example lesson plans for different time periods.

The running time of each segment is listed below:

Activities 6 & 8: 35 minutes

Activities 4 & 5: 45 minutes

Activities 2, 4, 6, &7: 1 hour

**Contents**

**Movie Segment 1: About HPV**

Activity 1: HPV Question Panel (14 minutes)

Activity 2: Crossword Puzzle (6 minutes)

**Movie Segment 2: HPV Vaccination**

Activity 3: Matching Game (5 minutes)

Activity 4: Educate the Public (30 minutes)

**Movie Segment 3: Decision Making**

Activity 5: Talk with your Parent (12 minutes)

Activity 6: The Decision-Making Process (10 minutes)

**Movie Segment 4: Practical**

Activity 7: Meditation Exercise (10 minutes)

**Movie Segment 5: Future**

Activity 8: Summary (12 minutes)

Activity 9: HPV Bingo (20 minutes)

**PART 1: ABOUT HPV TEACHER SUPPLEMENT**

**Activity 1: HPV Question Panel (14 minutes)**

Objective:

In this activity, students will develop and answer questions based on the film clip. This process will allow students to put the material into their own words, providing them with a better understanding of the information presented in the video.

Materials needed:

- Board for the teacher to write on.

Instructions:

Divide the class in half. Have each group come up with a team name and write them on the board **(1 minute)**. Then in their groups ask them to quietly write down three questions about HPV that they learned from the video clip; make sure the other team doesn’t hear! **(5 minutes)**.

Now, tell the class that each team will take turns asking and answering two of the questions they have just come up with. Note: The third question is just in case the teams come up with one of the same questions.

Designate a spokesperson to ask and to answer on each team (can be a different person for each question). Instruct the students that they will have one minute to talk in the group to come up with an answer. When the minute is up, have the spokesperson answer. Ask the other team if this is correct, and if it is, give them a tally below their team name. If something is missing or incorrect, be sure to point this out if the team is not able to do so. Switch teams and repeat so that each team has a chance to ask and answer two questions **(8 minutes)**.

**Sample questions and answers:**

1. **Q.** What does HPV stand for? **A.** Human papillomavirus.
2. **Q.** What are the two main diseases caused by HPV? **A.** Cervical cancer and genital warts.
3. **Q.** How is HPV transmitted? **A.** By human genital contact.
4. **Q.** Can you tell if someone has HPV and why/why not? **A.** No, sometimes there are warts present, but most of the time there are no symptoms.
5. **Q.** How is HPV related to cervical cancer? **A.** HPV can cause changes in the cells of the cervix (opening of the uterus/womb) that then lead to cancer.
6. **Q.** How common is HPV in Australia? **A.** About 80% of Australian women and men will get it at one point in their lives.
7. **Q.** Do condoms protect against HPV? **A.** Not always. They do reduce the risk, but you can still get HPV even if you use a condom.
8. **Q.** Where else can you get cancer on your body from HPV? **A.** Throat, anus, penis, vulva (lips of female genitals).

**Activity 2: Crossword Puzzle (6 minutes)**

Objectives:

Students will complete a crossword puzzle in this activity, which will provide a fast and easy way to review the material just viewed. This activity is most suitable if you do not have class time to devote to activity 1.

Materials needed:

- Copies of crossword puzzle found in appendix 1 for each student.

Instructions:

Tell the students to complete the crossword puzzle based on the film clip they just watched. Added fun: make it a competition!

Answer Key:

Across:

1. Abbreviation for the phrase: Human Papillomavirus. **Answer: HPV**

3. HPV is _____ by genital contact. **Answer: Transmitted**

5. HPV is the cause of all cases of this type of cancer. **Answer: Cervical**

7. HPV is very _____ in most countries. **Answer: Common**

9. Only _____ can get HPV. **Answer: Humans**

Down:

2. Other than cervical, anal or penile cancers, what other type of cancer can HPV cause? **Answer: Throat**

4. Sometimes there are no _____, so you can't always tell if you have HPV. **Answer: Symptoms**

6. These won't treat HPV like they will treat tonsillitis. **Answer: Antibiotics**

8. HPV causes changes to what in the body? **Answer: Cells**

10. HPV is a _____. **Answer: Virus**

11. People can get this to prevent certain types of HPV. **Answer: Vaccine**

12. The most common way people catch HPV. **Answer: Sex**

13. This protects against some types of sexually transmitted infection but does not provide full-proof protection against HPV. **Answer: Condom**

**PART 2: HPV VACCINATION TEACHER SUPPLEMENT**

**Activity 3: Matching Game (5 minutes)**

Objectives:

This activity will quickly reinforce for the students the important points the movie clip made. The cards should also clarify any confusion students might have had while watching the film.

Materials Needed:

- Two sets of cards printed from appendix 2. You may find it best to either laminate the cards or print on thick paper.

Instructions:

a) Divide class up into two teams. Give each team a set of the cards you printed face down. Tell the students that some cards have descriptions, and some cards have pictures. Based on the movie, students must match the descriptions to the pictures. Whichever team finishes first, wins! Make sure to verify that the cards are correct; if not, continue until one team gets them all correct.

b) Alternatively, the activity can be run with the whole class. Place the cards you printed face down. Tell the students that some cards have descriptions, and some cards have pictures. Based on the movie, students must match the descriptions to the pictures. Make sure to verify that the cards are correct.

Answer Key:

1. HPV virus-like particles, not the *actual* HPV virus (picture: virus-like particle)

2. Antibodies, or little warriors that will kill the HPV virus (picture: antibody)

3. Three doses of the HPV vaccine are needed (picture: vaccines)

4. Sometimes you will feel some pain or swelling with the HPV vaccine (picture: student’s arm)

5. Sometimes you may feel some aches and pains with the HPV vaccine (picture: aching people)

6. This picture shows how HPV is transmitted (picture: doctor’s chart with genitals highlighted)

7. One reason to get the vaccine when you are younger is that your body creates more antibodies after you get the vaccine (picture: lots of antibodies on left; fewer on right)

8. Some of us can get and transmit HPV, but not cervical cancer (picture: boy)

**Activity 4: Educate the Public (30 minutes)**

Objectives:

In this activity, have the students work in teams to put into their own words what they have learned from the movie clip about the HPV vaccine. They should provide a well-rounded factual explanation of the HPV vaccine without providing their own opinions.

Alternatively, if you have less time to spare on this activity, students can break into small groups and develop a "brain storm" about HPV. Each group can then deliver a short presentation about their "brain storm."

Materials Needed:

- 5 poster boards, one for each group
- 5 copies of the image print-outs from the movie (appendix 3)
- Markers
- Tape
- Scissors (or you may pre-cut the images)

Instructions:

Divide the class up into 5 groups. Ask them to create a poster that will educate the public about the HPV vaccine. They should consider who they are addressing their posters to: parents, students (boys or girls or both), teachers, etc. Ask them to try to make their posters appropriate for that audience through pictures and language **(20 minutes)**.

- Describe HPV: What are the symptoms? Who is most susceptible? Is it caused by a virus or bacteria?
- Who should be vaccinated, and when? How often should a person be vaccinated?
- How does the vaccine work?
- What are some possible side effects of the vaccine?
- Are there any other interesting facts about this vaccine?

Have each team present their poster for one minute. Have each group concentrate on explaining one of the above bullet points in their presentation **(10 minutes)**. Hang the posters around the classroom when finished.

Adapted from: <http://www.discoveryeducation.com/teachers/free-lesson-plans/common-vaccinations.cfm>

**PART 3: DECISION-MAKING**

**TEACHER SUPPLEMENT**

**Activity 5: Talk with your Parent (12 minutes)**

Objectives:

In this activity, students will reflect on the possibility of discussing the HPV vaccination to their parents to talk about making a decision when they have all the facts. They will develop strategies to discuss vaccination with a view to making the decision together with their parents or guardian/s, although their caregiver ultimately has the final say.

Materials Needed:

None.

Instructions:

Divide the students into groups of 3. Tell the class that one student in the group will pretend to be the parent, one the student, and one the observer **(2 minutes**).

Ask the groups to act out a scenario where the student is talking to the parent about getting the HPV vaccination. The parent and student may choose whether they are for or against getting the vaccine, but reasons should be given in the discussion. Instruct the observer to pay attention to how the student brings up the conversation, how the parent reacts, and what went well, or what could be improved **(4 minutes)**.

If students need assistance developing their role-play scenario, you could assist with the following questions to guide the development of the scenario:

What view do you think your parents will have about HPV vaccination?

Is this the same view that you think you have now you know all the facts?

What would your parents need to make a decision?

What are some of the hardest topics to discuss with your parents? Would HPV vaccination be one of them?

Have you got any strategies for good and bad times to bring the subject up?

You could also provide students with various scenarios to play out and consider: e.g. separated parents who have different views about vaccination.

Next, ask the observer in each group to report back to the class. Allow the “actors” in the group to contribute as well if the observer is stuck **(6 minutes)**.

Class debrief led by teacher:

- How did the student bring up the conversation?
- What were the positive strategies used in the conversation?
- Things that could possibly be improved?
- Was it different for boys than girls?
- Were there challenges in your particular family?

**Activity 6: The Decision-Making Process (10 minutes)**

Objectives:

In this activity, students will learn that respectfully talking with their parents in a positive and respectful way and having all the important information are the two most important factors when deciding whether or not to get the HPV vaccine.

Materials:

- Plain sheet of paper and pencil for each student

Instructions:

Hand the students a plain sheet of paper and ask them to fold it into horizontal thirds. Have the students write ‘pros’ on the top third, ‘cons’ on the middle third, and ‘discussion’ on the bottom.

Ask the students to write pros and cons (at least 3 for each) of getting the HPV vaccine on each part of their paper **(2 minutes).**

Then, based on the pros and cons they have listed, ask them to think about things they might discuss with their parents about the HPV vaccine and how they would do so. This could be a list of questions they might ask their parent, statements of how they would bring up the conversation, concerns they might want to bring up with their parent, or their own opinions they want to share with their parent **(3 minutes)**.

Ask for students to share their ideas they just brainstormed for ways to talk with their parents about HPV vaccination **(5 minutes)**.

Examples:

*Pros*

- I will be protected from the 4 types of HPV
- I will be less likely to get cervical cancer (or other genital cancers)
- I will be less likely to get genital warts
- I will be less likely to pass HPV along to someone else
- It is offered in schools for free in Year 7 (or 8 in varying states)

*Cons*

- The vaccine might hurt or feel weird
- I could faint or cry
- I could have a side effect like a sore arm, or fever
- I am concerned that the vaccine is still so new, I want to know about the long-term effectiveness and any long-term side effects

*Discussion*

- Could ask parent:
  - Do you think I should get the HPV vaccination? Why/why not?
  - Do you have any questions about the HPV vaccine?
  - Can we talk about this together with a doctor?
- Could tell parent:
  - I have learned that it’s important to have the vaccine before becoming sexually active, and it actually works better in younger people.
  - Let’s look at my educational materials together and talk this over.
  - I do or do not want to get the vaccine; here’s why I feel this way.

**PART 4: PRACTICAL TEACHER SUPPLEMENT**

**Activity 7: Meditation Exercise (10 minutes)**

Objectives:

In this activity, students will learn to practice deep breathing techniques as part of stress reduction before vaccination.

Materials needed:

None.

Instructions:

Introduce the concept of deep breathing as a stress reduction strategy that can be used on vaccination day, or when they feel stressed in other situations.

Ask the students to be seated in a chair. Read the directions below to students taking special care to read very slowly and speak in a relaxed tone:

1. Sit in a chair straight and tall with feet hip distance apart on the floor.
2. Allow your arms and hands to fall at your sides relaxed.
3. Concentrate on making sure your entire body is relaxed.
4. If you feel comfortable, close your eyes. If not, softly gaze at a spot in front of you.
5. Focus on your lower belly and imagine a small balloon in that space. Exhale all of your air out, as you put a hand on your lower belly. Keep your hand there.
6. Breathe in for four counts through your nostrils. (pause) At the same time, imagine the balloon is inflating…. sloooowly growing.
7. Hold the breath in for four counts. (pause for 3 seconds)
8. Exhale slowly through the nostrils for four counts. (pause) Imagine the balloon gently deflating; shrinking.
9. Now, hold the breath out for four counts. (pause for 3 seconds)
10. Keep your hand over the lower belly to feel it go up and down as we continue.
11. Repeat steps six through nine 5-8 times.

Ask students how their bodies feel after the exercise. (Are they more relaxed/calm? Do they feel lighter? Happier? Sleepier?) Tell the students that they can do this while waiting in line to be vaccinated to help them feel calmer.

**PART 5: FUTURE TEACHER SUPPLEMENT**

**Activity 8: Summary (12 minutes)**

Objectives: In this activity, students will summarize one of the five broad topics covered in the video as a group and then present their summary points to the class.

Materials:

- 5 sheets of paper
- 5 pencils

Instructions:

Write the five topic headings on slips of paper: About HPV, HPV Vaccination, Decision-making, Practical things for the day of the vaccination, and Future. Separate students into 5 groups and hand each a slip of paper. Ask the students to talk in their groups to come up with as many summary points as they can for each topic (**5 minutes**).

When the students are finished, or after they have brainstormed for 5 minutes, ask each group to list off their summary points. If a group needs help, ask the class some of the questions below **(7 minutes)**.

1. About HPV
   - What is HPV? *Human papillomavirus, a virus than can cause cervical cancer and genital warts.*

- How is HPV transmitted? *From person-to-person genital contact.*
- Can you tell if someone has HPV? *You can’t! Sometimes you can see warts, but most of the time there are no symptoms.*
- How is HPV related to cervical cancer? *HPV can cause changes in the body’s cells that then lead to cancer.*

1. HPV Vaccination

- What does the HPV vaccine protect against? *Cervical cancer, other genital cancers, and genital warts.*
- How does it work? *In a series of 3 shots, the vaccine injects particles that look like HPV, so the body can learn what HPV looks like and create antibodies to prevent an actual HPV infection.*
- Can you get HPV from the vaccine? *NO, the vaccine does not actually contain HPV, only particles that look like it.*
- Are there any side effects? *Yes, there can be: pain, swelling, redness at the injection site, you may feel aches and pains in your body for a few days.*
- Why do boys get the vaccine?  *Boys can get HPV too. Getting the HPV vaccine will prevent them from getting genital warts, and also keep them from passing it along to girls.*

1. Males and HPV

- Can you tell me more about what happens when males get HPV? *Males can get HPV just as commonly as females. This means that most males will catch HPV at some point in their lifetime.*
- How do males know if they have HPV?
- What diseases can males get from HPV? *Genital warts, early or pre- cancer, and rarely cancer. In males, HPV can also rarely cause cancer of the throat, penis, and the anus. Females can also rarely get pre-cancer and cancer of the throat and anus.*
- Does that mean that I could get cancer from HPV? *Both males and females can get cancer from HPV, although it is very uncommon.*
- What is the best way to prevent HPV infection? *The most effective way to prevent genital warts and HPV related cancers is to have the HPV vaccine.*
- Is the vaccine just as effective for both boys and girls? *Yes. The vaccine protects against 90% of genital warts and at least 70% of genital and anal cancers for females and males.*
- Decision-making
- How can you make a decision about whether to be vaccinated? *You’ll need to talk to your parents and decide together. Learn as much as you can about the vaccine, and encourage your parents to do the same.*
- How can you talk to your parents about HPV vaccination? *Talk to them about what you’ve learned. Say it’s best to have it early, before sexual activity, and that it actually works better in the body at an early age.*
- Practical things for the day of the vaccination
  - Is there anything you need to do before you get vaccinated? *Nothing, other than try to eat a good breakfast and relax!*
  - Does the HPV vaccination hurt? *It depends on the person. It may sting or feel chilly, but you can distract yourself so that you don’t feel it as much.*
  - What are some things you can do to help if you are afraid of needles*? Remain calm! Breathe in while slowly counting to 4, breathe out while slowly counting to 6. You can also hum a song while patting your leg, or think of things you are learning for a test.*
- Future
- What will the HPV vaccine protect you against in the future? *You will be protected from the four types of HPV that cause most genital warts and cervical cancers. You will not be protected against other sexually transmitted infections or unwanted pregnancy. And, when girls are older, they’ll still need to have regular Pap smears because the vaccine does not protect against all cancer-causing HPV types.*
  - What is a pap smear? *Given to girls usually every two years after they become sexually active (or at 18) to check for changes in the cells on the cervix.*

**Activity 9: HPV Bingo (20 minutes)**

Objective**:** Students’ bingo boards have answers to HPV questions. As the instructor reads out the questions, they will cover the answer on their board if they have it.

Materials:

- - Print out a bingo board for each student (see appendix 4).
  - Bingo chips: Laminate a thick coloured piece of paper and use a paper cutter to cut small squares, or use counters or coins.

Instructions:

Pass out the boards to each of the students. Now, tell the students that you will be reading out descriptions, and they will need to guess the word. Once they have guessed the word correctly, tell them they may put a chip on their board if they see this word. The first person to get 4 in a row should call out bingo!

1. What does HPV stand for? **Human papillomavirus**
2. What does HPV cause in only women? **Cervical Cancer**
3. What does HPV cause in both men and women? **Genital warts**
4. Why can’t you tell if someone has HPV or not? **No symptoms**
5. What method of prevention does not always protect you from getting HPV? **Condom**
6. Other than the genitals, where else can you get cancer from HPV? **Throat**
7. How many doses is the vaccine given in? **3**
8. Little warriors that will kill any HPV virus that may try to infect your body are: **Antibodies**
9. Who must you speak with to decide if the HPV vaccine is right for you? **Parents**
10. What can you do if you are afraid of needles, or nervous on the day? **Relaxation exercises**
11. What do girls still need to do regularly they have been vaccinated? **Pap smears**
12. How is HPV transmitted? **Genital contact**
13. What percent of women in Australia have HPV? **80**
14. What type of particles are in the HPV vaccine? **virus-like**
15. What other sexually transmitted infections does the vaccine protect against other than HPV? **None**
16. What does HPV change in the body that makes people get cancer? **Cells**
17. What does the body learn about the HPV virus from the vaccine so that it can prevent infection? **Shape**
18. Ideally, you should get the HPV vaccine before you are what? **Sexually active**
19. In the rare case that someone has an allergic reaction to the vaccine, who is there to treat it right away? **Nurse**
20. You will have a higher level of protection from the vaccine if you get it when you are: **Young**
